# Uncharacterized RNAs in Plasma of Alzheimer’s Patients Are Associated with Cognitive Impairment and Show a Potential Diagnostic Power

**DOI:** 10.3390/ijms21207644

**Published:** 2020-10-15

**Authors:** Cristina Barbagallo, Maria Teresa Di Martino, Margherita Grasso, Maria Grazia Salluzzo, Francesca Scionti, Filomena Irene Ilaria Cosentino, Giuseppe Caruso, Davide Barbagallo, Cinzia Di Pietro, Raffaele Ferri, Filippo Caraci, Michele Purrello, Marco Ragusa

**Affiliations:** 1 Department of Biomedical and Biotechnological Sciences, Section of Biology and Genetics G. Sichel, University of Catania, 95123 Catania, Italy; barbagallocristina@unict.it (C.B.); dbarbaga@unict.it (D.B.); dipietro@unict.it (C.D.P.); purrello@unict.it (M.P.); mragusa@unict.it (M.R.); 2 Department of Experimental and Clinical Medicine, Magna Graecia University, 88100 Catanzaro, Italy; teresadm@unicz.it (M.T.D.M.); fscionti20@gmail.com (F.S.); 3 Oasi Research Institute—IRCCS, 94018 Troina, Italy; grassomargherita940@gmail.com (M.G.); msalluzzo@oasi.en.it (M.G.S.); fcosentino@oasi.en.it (F.I.I.C.); forgiuseppecaruso@gmail.com (G.C.); rferri@oasi.en.it (R.F.); 4 Department of Drug Sciences, University of Catania, 95125 Catania, Italy

**Keywords:** AD, non-coding RNAs, lncRNAs, biomarkers, non-invasive diagnosis

## Abstract

Alzheimer’s disease (AD) diagnosis is actually based on clinical evaluation and brain-imaging tests, and it can often be confirmed only post-mortem. Therefore, new non-invasive molecular biomarkers are necessary to improve AD diagnosis. As circulating microRNA biomarkers have been proposed for many diseases, including AD, we aimed to identify new diagnostic non-small RNAs in AD. Whole transcriptome analysis was performed on plasma samples of five AD and five unaffected individuals (CTRL) using the Clariom D Pico Assay, followed by validation in real-time PCR on 37 AD patients and 37 CTRL. Six differentially expressed (DE) transcripts were identified: GS1-304P7.3 (upregulated), NONHSAT090268, TC0100011037, TC0400008478, TC1400008125, and *UBE2V1* (downregulated). Peripheral blood mononuclear cells (PBMCs) may influence the expression of circulating RNAs and their analysis has been proposed to improve AD clinical management. Accordingly, DE transcript expression was also evaluated in PBMCs, showing no difference between AD and CTRL. ROC (receiver operating characteristic) curve analysis was performed to evaluate the diagnostic accuracy of each DE transcript and a signature including all of them. A correlation between cognitive impairment and GS1-304P7.3, NONHSAT090268, TC0100011037, and TC0400008478 was detected, suggesting a potential association between their extracellular abundance and AD clinical phenotype. Finally, this study identified six transcripts showing altered expression in the plasma of AD patients. Given the need for new, accurate blood biomarkers for AD diagnosis, these transcripts may be considered for further analyses in larger cohorts, also in combination with other biomarkers, aiming to identify specific RNA-based biomarkers to be eventually applied to clinical practice.

## 1. Introduction

Alzheimer’s disease (AD) is a neurodegenerative disorder and a leading cause of dementia in the elderly, accounting for 60–80% of all dementia cases worldwide. It is caused by the aggregation of misfolded proteins leading to the formation of extracellular amyloid plaques and intracellular neurofibrillary tangles of hyperphosphorylated tau protein [1]. To date, AD diagnosis is based on evaluation of cognitive symptoms (memory loss, disoriented behavior and impairments in language, comprehension, and spatial skills), combined with neuroimaging and neuropsychological markers [2]. Neuropsychiatric symptoms, such as depression, psychosis and agitation, are also frequent in people with AD and represent a common cause of hospitalization [3]. AD diagnosis can often be confirmed only though post-mortem analysis of brain tissues [4]. For this reason, research on appropriate diagnostic biomarkers is of paramount importance for a non-invasive diagnosis of AD and it has been very active since the early 2000s. The most important features for diagnostic biomarkers are not only sensitivity and specificity, making them able to discriminate affected from unaffected individuals with high accuracy, but also the low cost and the ease of sample collection and analysis. The most accurate biomarkers currently available for AD diagnosis are retrieved in cerebrospinal fluid (CSF), where it is possible to dose amyloid beta (Aβ), tau protein, and phosphorylated tau protein [5]. The great advantage of these biomarkers is their high sensitivity and specificity, likely due to the detection in CSF, which is in direct contact with the diseased brain. Unfortunately, the downside is that CSF collection is an invasive and expensive procedure. Therefore, researchers are actively working to identify new biomarkers that are easier to collect. The most common source of biomarkers is blood (specifically, serum, or plasma), as it can be collected easily and at low cost. Notably, blood-based biomarkers represent a novel tool to identify individuals at an early stage of AD [6]; indeed, in the last few decades, several blood circulating molecules have been investigated as AD diagnostic biomarkers, including AD-related molecules (Aβ, tau and phosphorylated tau), proteins, metabolites, and microRNAs (miRNAs). However, several reports showed promising results that have not been confirmed in independent studies [7]. The application of circulating RNA molecules, including non-coding RNAs (ncRNAs), as biomarkers has been widely explored in the last few decades; miRNAs have been suggested as diagnostic biomarkers for several diseases, including neurodegenerative [8,9], neurodevelopmental [10], traumatic [11], neoplastic [12,13], and metabolic conditions [14,15]. Indeed, miRNAs have been detected in all analyzed biological fluids [16,17,18], making them useful as disease biomarkers measurable in all body districts. Similarly, long non-coding RNAs (lncRNAs) and circular RNAs (circRNAs), a recently discovered class of ncRNAs, are emerging as new potential disease biomarkers, also in AD [19]. The exploitation of RNA molecules as diagnostic biomarkers would represent a great advantage for clinical practice: in fact, RNA is very easy to isolate and can be analyzed with high accuracy by common molecular biology techniques.

It has recently been proposed that immune system activation and neuroinflammation may also play an important role in AD pathogenesis [20]. Inflammatory processes have been observed in association with AD, and a colocalization between AD lesions and pathogen signatures has been reported. Moreover, in vivo models showed that deposits of Aβ and tau are induced by infections with both viruses and bacteria [21]. Given this association between AD pathogenesis and the immune system, several studies on immune cells in AD are emerging. Peripheral blood mononuclear cells (PBMCs) and lymphocytes have been proposed for their potential diagnostic value, because they are affected in AD patients and can reflect in the periphery biological alterations found in the central nervous system (CNS) [22,23]. Moreover, immune cells reside in blood and represent one of the most abundant cell types in this compartment of the body. Therefore, it may be hypothesized that differential expression of RNA-based biomarkers detected in blood could be influenced by expression levels of the transcripts detected in PBMCs.

Because of these reasons, the aim of this study was to identify new potential RNA-based biomarkers in plasma for AD diagnosis. Furthermore, we investigated the potential contribution of immune cells to the dysregulation of RNA molecules in the plasma of AD patients compared to unaffected individuals.

## 2. Results

### 2.1. Transcriptome Analysis of Plasma Samples of AD Patients Compared to Unaffected Individuals

**Figure 1:**
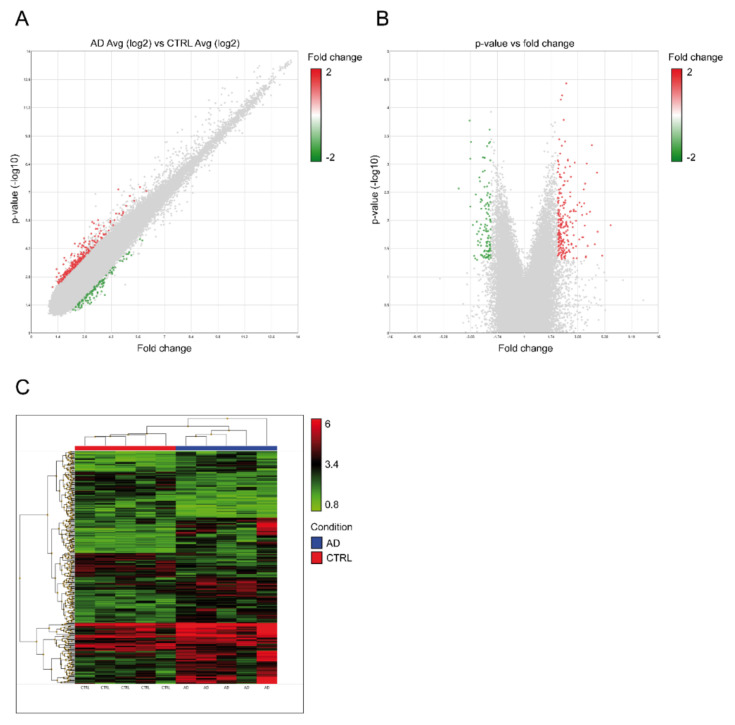
Results of microarray profiling. (**A**) scatter plot showing fluorescence intensity of significantly deregulated transcripts; (**B**) volcano plot showing significantly deregulated transcripts; (**C**) hierarchical clustering of analyzed samples. Legend: (**A**) and (**B**) colored dots show significantly deregulated transcripts (*p* < 0.05), with red representing upregulation (fold change > 2) and green representing downregulation (fold change < −2); (**C**) fluorescence intensity data are plotted. AD: Alzheimer’s disease patients; CTRL: unaffected individuals.

The whole transcriptome analysis performed using the Clariom D Pico assay investigated the expression of more than 540,000 transcripts and identified RNA molecules showing a differential expression between AD patients and unaffected individuals (CTRL). Microarray data were analyzed both considering the five AD-CTRL pairs and comparing the AD group with the CTRL group (paired and unpaired analyses, respectively). Results were filtered selecting only the transcripts showing a high fluorescence intensity in the microarray. We focused our attention on DE transcripts common to paired and unpaired results, filtered according to *p*-value and fold change, obtaining a total of 325 deregulated transcripts, of which 215 were upregulated and 110 were downregulated (Figure 1).

### 2.2. Validation of Microarray Results by Real-Time PCR

DE transcripts identified through microarray profiling were successively validated in an independent cohort of 74 individuals, including 37 AD patients and 37 unaffected controls, matched for sex and age. We selected 48 transcripts from profiling results, showing the highest fluorescence intensity and the strongest *p-*values and fold changes. First of all, the expression of these transcripts was evaluated in a small cohort of plasma samples (data not shown) to assess their amplificability in PCR; accordingly, validation analysis was performed on 19 transcripts showing detectable levels in Real-Time PCR. Validation assays confirmed the differential expression of 6 transcripts, namely GS1-304P7.3, NONHSAT090268, TC0100011037, TC0400008478, TC1400008125, and *UBE2V1*. For all transcripts, both paired and unpaired analyses showed statistical significance according to both endogenous controls. In particular, GS1-304P7.3 showed increased expression in the AD group compared to the CTRL group, while NONHSAT090268, TC0100011037, TC0400008478, TC1400008125, and *UBE2V1* levels were reduced in AD plasma samples compared to the CTRL group (Table 1).

Statistical analyses performed to evaluate age and sex as confounding factors showed no significant results, confirming that these parameters do not represent confounding factors in our study (data not shown).

We also investigated the existence of any difference in DE transcript expression associated with gender. This analysis was performed within each pathological group (AD males vs females and CTRL males vs. females) and within individuals of the same gender (female AD vs CTRL and male AD vs CTRL): no statistical difference was observed.

### 2.3. Evaluation of Diagnostic Accuracy through ROC Curves

**Figure 2:**
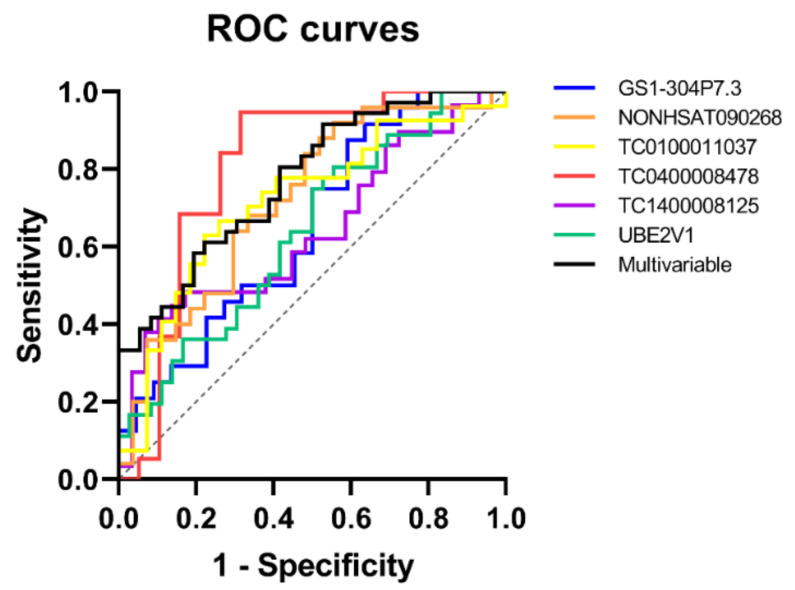
Univariable and multivariable ROC curves computed on plasma expression levels of differentially expressed (DE) transcripts.

The identification of differentially expressed (DE) transcripts in plasma of AD patients compared to unaffected individuals may pave the way to potential diagnostic applications of these transcripts in clinical practice. For this reason, we computed receiver operating characteristic (ROC) curves to assess the potential diagnostic accuracy of DE transcripts. First of all, we evaluated the accuracy of each DE transcript, considered as an individual plasma biomarker for AD. Univariable ROC curves are shown in Table 2 and Figure 2.

Results showed significant curves for GS1-304P7.3, NONHSAT090268, TC0100011037, TC0400008478, and *UBE2V1*, with a good diagnostic performance for TC0400008478 (AUC > 0.8) and a fair performance (AUC > 0.7) for GS1-304P7.3, NONHSAT090268, and TC0100011037.

Moreover, we evaluated the diagnostic accuracy of a biomarker signature including all six DE transcripts; this multivariable ROC curve showed a significant *p-*value but did not improve the diagnostic performances of single univariate univariable ROC curves (Table 3 and Figure 2).

### 2.4. Peripheral Blood-Isolated Cell Expression Analysis

Expression of DE transcripts was also evaluated in PBMCs obtained from AD patients and age-and sex-matched unaffected individuals. Although real-time PCR results showed an expression trend similar to that reported in plasma (except for GS1-304P7.3), no statistically significant difference was observed (Table 4).

### 2.5. Correlation with Clinical and Cognitive Phenotypes

Plasma levels of DE transcripts and clinicopathological parameters measuring cognitive impairment and its progression were correlated. A significant correlation between Mini Mental State Examination (MMSE) T0 and four out of the six DE transcripts was observed: in particular, a negative correlation was observed between MMSE score at T0 and GS1-304P7.3, the only transcript showing increased expression in AD patients, while NONHSAT090268, TC0100011037, and TC0400008478, downregulated in the plasma of AD patients, showed a positive correlation with MMSE T0. No significant correlation was observed between Delta MMSE/month and any transcripts (Table 5).

## 3. Discussion

Blood-based biomarkers will become essential in the near future to perform non-invasive diagnosis of AD. According to this scenario, RNA-based diagnostic biomarkers may represent a novel tool to improve the diagnosis of AD. The aim of this study was to perform a transcriptome analysis of plasma from mild to moderate AD patients and unaffected individuals, in order to identify RNA transcripts (including mRNAs, lncRNAs and circRNAs) to be applied as new potential diagnostic biomarkers. Our cohort included AD patients and unaffected individuals matched for sex and age, in order to exclude the influence of these features on the transcriptome. Through microarray analysis and subsequent validation in real-time PCR, we identified six transcripts showing differential expression; in particular, GS1-304P7.3 was upregulated in the AD group compared to the CTRL group, while NONHSAT090268, TC0100011037, TC0400008478, TC1400008125, and *UBE2V1* showed reduced expression in AD plasma samples compared to the CTRL group. The same expression alteration was confirmed by both paired and unpaired analyses, showing that sex and age did not influence transcript expression in our cohort, suggesting the potential strength of these biomarkers. It has recently been hypothesized that immune system activation plays a key role in AD pathogenesis [20]. The key contribution of inflammation in AD pathology was hypothesized more than twenty years ago and recent studies have demonstrated that this early disease-aggravating CNS inflammation starts decades before the presentation of severe cognitive impairments or AD [24]. The potential role of the immune system cells may be enhanced by their abundant presence in blood, where they represent one of the principal cell types: indeed, altered expression of ncRNAs in immune cells may influence expression profiles of the same ncRNAs circulating in the blood. For these reasons, we also investigated the expression of these transcripts in PBMCs isolated from a sub-cohort of AD patients and unaffected individuals. However, this analysis showed no significant differential expression within PBMCs. Further studies are needed in larger cohorts of AD patients.

Among the transcripts identified here as differentially expressed in the plasma of AD patients compared with unaffected individuals, *UBE2V1* is the only protein-coding and characterized one. *UBE2V1* (ubiquitin conjugating enzyme E2 V1) encodes a ubiquitin-conjugating enzyme involved in the proteasome degradation pathway. A recent study showed that *UBE2V1* promotes protein aggregation in rat cardiomyocytes [25]; this report could be relevant for a potential involvement of *UBE2V1* in AD, where protein aggregation and amyloid deposition is one of the histopathological hallmarks. Moreover, reduced *UBE2V1* levels were observed in the cerebellum and blood of a mouse model of Rett syndrome, a rare neuropsychiatric disorder [26]. No data are available to date on humans, but reports in animal models suggest that *UBE2V1* plays a key role in synaptic plasticity and function by affecting postsynaptic density-95 (*PSD95*) scaffolding properties [27]. We found a reduction of *UBE2V1* in the plasma of AD patients. This result would suggest that *UBE2V1* might be analyzed in the near future as a novel pharmacological target in AD. The other DE transcripts are uncharacterized, and very scarce data are available in the literature. By blasting sequence transcripts, we investigated their position within the genome, aiming to infer some information about their putative functions. These transcripts could act as regulators of expression of the neighbor genes by binding regulatory sequences, such as silencers/enhancers [28], or as splicing regulators of host genes when their sequences overlap [29]. Therefore, by mapping DE transcripts on the genome, we could speculate on their indirect involvement in AD pathogenesis, performed by regulating the expression of neighbor genes involved in the disease. GS1-304P7.3 (TC0100010930) maps on chromosome 1, in an intergenic region between the lncRNA *PDC-AS1* (about 600 bp upstream) and the locus of *PTGS2* (about 149 Kb downstream). GS1-304P7.3 is known to be expressed in endothelial cells, suggesting its involvement in angiogenetic processes [30]. A link between AD and angiogenesis was previously reported, showing a direct involvement of endothelial cells in the neovascularization process occurring in AD brain [31,32]. No information is available on *PDC-AS1* (PDC antisense RNA 1), but the function of its sense transcript *PDC* (phosducin) has been described. The exact function of antisense transcripts has not been elucidated, but evidence suggests the existence of a mutual post-transcriptional regulation involving sense and antisense transcripts [33]. *PDC*, encoding a phosphoprotein involved in the process of vision in the retina, has been associated with stress-induced hypertension [34,35]. Hypertension, in turn, has been associated with an increased risk to develop AD [36,37]. *PTGS2* (prostaglandin-endoperoxide synthase 2) encodes the cyclooxygenase enzyme, involved in the biosynthesis of prostaglandins; different single nucleotide polymorphisms in *PTGS2* have been associated with AD, resulting in protection or increased risk of developing the disease [38,39]. In vivo experiments in mouse models showed that inhibition of *PTGS2* expression is increased by Aβ deposition, leading to inflammation [40], while inhibition of the enzyme prevents memory deficits [41]. TC0100011037 maps on an intergenic region in chromosome 1, between the lncRNA *LINC01031* (about 100 kb upstream) and the pseudogene *RPL23AP22* (about 286 kb downstream). No information is available in the literature on both the neighbor genes. However, *RPL23AP22* is a pseudogene of *RPL23A* (ribosomal protein L23a), a ribosomal protein that has been associated with AD pathogenesis in transgenic models [42,43]. TC0400008478 maps on chromosome 4, between the genetic loci of *ARSJ* (about 269 kb upstream) and *UGT8* (about 344 kb downstream). *ARSJ* (arylsulfatase family member J) encodes a sulfatase enzyme that has not been associated with AD to date. *UGT8* (UDP glycosyltransferase 8) encodes a UDP-glycosyltransferase involved in the biosynthesis of galactocerebrosides, sphingolipids abundant in the central nervous system [44]. *UGT8* has been reported as a key gene in a mouse model of AD, where its genetic ablation induced the dysregulation of the same myelination pathways altered in human AD brain [45]. Moreover, *UGT8* expression was shown to increase with cognitive impairment in human brain [46]. TC1400008125 maps on chromosome 14, within a 37-kb intron of the lncRNA *LINC02299*. No information is available on the function of this lncRNA. NONHSAT090268 (TC0300007694) maps on chromosome 3, within a 141-kb intron of the lncRNA *ADAMTS9-AS2*. *ADAMTS9-AS2* (ADAMTS9 antisense RNA 2) is an antisense lncRNA with unknown function, recently reported as a tumor suppressor gene in glioma [47] and associated with senile neurodegeneration and AD [48]. Its sense transcript, *ADAMTS9* (ADAM metallopeptidase with thrombospondin type 1 motif 9), encodes for a disintegrin and metalloproteinase with thrombospondin motifs enzyme; single nucleotide polymorphisms in *ADAMTS9* sequence have been associated with cognitive aging [49]. *ADAMTS9* suppresses β-catenin and the canonical Wnt signaling pathway [50] that is known to be inhibited in the AD brain [51,52].

Hypothesizing a potential application as diagnostic plasma biomarkers for these transcripts, we evaluated their diagnostic performance by computing ROC curves. We computed two types of ROC curves, aiming to identify the best biomarker or combination of biomarkers, creating a signature specific for AD diagnosis. It is now widely proven that a signature including multiple biomarkers may show higher sensitivity and specificity than a single one. Univariable ROC curves were computed considering each biomarker by itself in order to test the diagnostic accuracy of the single transcript. We obtained significant curves for GS1-304P7.3, NONHSAT090268, TC0100011037, TC0400008478, and *UBE2V1*, but only the curve built on TC0400008478 reached a good diagnostic performance (AUC > 0.8). Similarly, a signature including all six biomarkers did not increase diagnostic performance. To date, the best diagnostic performance for AD has been reported for biomarkers detected in CSF [53]. This observation is not surprising, since CSF is in direct contact with brain interstitial fluid; therefore, biomarkers in CSF are specific of the brain and not diluted by others originating from different body districts (two conditions that characterize biomarkers detected in blood). Unfortunately, CSF sampling is invasive for patients and economically expensive, making blood-based biomarkers, even with their disadvantages, more attractive. According to their dilution in the circulation, blood biomarkers showed a lower diagnostic performance in different reports: a recent study analyzed the diagnostic accuracy of total Aβ42-to-Aβ40 ratio and free Aβ42-to-Aβ40 ratio in plasma, showing AUC values of 0.775 and 0.710, respectively [54]; another study reported the diagnostic performances of Aβ1–42, and of the ratios of Aβ1–42 to a novel APP669–711 fragment (APP669–711/Aβ1–42) and Aβ1–40/Aβ1–42, analyzed in different cohorts of patients, resulting in ROC curves with variable AUCs [55]. Diagnostic performance of DE transcripts here identified conforms with Aβ-based blood biomarkers currently reported in the literature. However, it is important to underline that the diagnostic power reported for a specific biomarker is very difficult to validate in a different study. This difficulty could be explained by the diversity of the enrolled cohort (age, ethnicity, diagnostic criteria), the different procedure and technique applied for biomarker detection and measurement, the different lab equipment used. Therefore, the best and only way to compare diagnostic accuracy of DE transcripts and previously proposed biomarkers would be to perform a dedicated study, where all different biomarkers are measured in the same patients with the same workflow and technique. Looking for the best diagnostic performance, it may be useful to combine RNA-based (including both small and long non-coding RNAs) and Aβ-based biomarkers from plasma to assess the diagnostic accuracy of an AD specific signature including both types of biomarkers. Further studies are needed to investigate the efficacy of such signatures in large and multicentric cohorts of patients.

Correlation analysis showed that four out of the six DE transcript are associated with cognitive impairment. In particular, GS1-304P7.3 showed a negative correlation with MMSE score, while NONHSAT090268, TC0100011037, and TC0400008478 showed a positive correlation with global cognitive function. These opposite correlation trends are in agreement with the opposite deregulation trends observed in plasma, where GS1-304P7.3 was the only transcript with increased levels in AD patients, while the other RNA-based biomarkers showed a reduced expression. This association with cognitive impairment suggests an active role of these transcripts in AD pathogenic processes that might be better examined in animal models of AD.

## 4. Materials and Methods

### 4.1. Patient Recruitment and Plasma Sample Processing

This study was approved by the Ethics Committee of Oasi Research Institute—IRCCS, Troina (Italy) (20 March 2019; Code: 2019/03/18/CE-IRCCS-OASI/18) and it was performed in accordance with the Declaration of Helsinki. A total of 84 participants, consisting of 42 AD mild-moderate patients and 42 CTRL, were included in this study. AD patients were recruited at the Oasi Research Institute—IRCCS, Troina (Italy) (Table 6), where the NINCDS-ADRDA criteria (1984) for AD was used [56] in combination with DSM-IV guidelines. Written informed consent was obtained from all the participants or their families. Unaffected individuals matched for age and sex, with no cognitive impairment and family history of AD or Vascular Dementia, were selected. Total cognitive function assessment was carried out by using the Mini-Mental State Examination (MMSE) test [57]. For AD patients, total cognitive function was measured at both baseline (T0) and after 6–24 months (T1). Delta MMSE/month, which represents the progression rate of cognitive decline [58], was calculated as follows:

(MMSE score at T1−MMSE score at T0)/Interval between follow-up visit (months)

For plasma isolation, blood samples were collected in vacutainer tubes and centrifuged at 1800× *g* for 15 min at 20 °C; plasma was stored at −80 °C until analysis.

### 4.2. RNA Isolation from Plasma Samples

Total RNA was isolated from plasma samples using the miRNeasy Mini-Kit (Qiagen, Hilden, Germany), according to the Qiagen supplementary protocol for total RNA isolation from serum and plasma. RNA was finally eluted in 200 µL RNase-free water and then precipitated by adding 3M sodium acetate (pH 5.2), 100% ethanol, and 1 µg glycogen as carrier. After overnight incubation at −80 °C, samples were centrifuged (12,000× *g*, 30 min, 4 °C) and pellets were washed twice in 75% ethanol. The final RNA pellet was dissolved in RNase-free water. RNA quantification was performed by Nanodrop One (Thermo Fisher Scientific, Waltham, MA, USA).

### 4.3. Microarray Analysis

Whole transcriptome analysis of plasma was performed in 5 AD patients and 5 matched controls by using Clariom D Pico Assay (Thermo Fisher Scientific, Waltham, MA, USA). This technology analyzes the expression of more than 540,000 coding and non-coding transcripts, including mRNAs, circRNAs, lncRNAs, miRNA precursors and other small RNAs, loading a low input of total RNA. Briefly, 10 ng of total RNA were retrotranscribed in single-stranded cDNA containing T7 promoter sequence at the 5′ end. 3′ Double-stranded cDNA was synthesized by adding an adaptor as a template; pre-IVT amplification reaction was optimized with 12 cycles of amplification, as previously reported [59]. The double-stranded DNA was used as a template for antisense RNA synthesis and overnight amplification (14 h) by in vitro transcription (IVT), using T7 RNA polymerase. Approximately 20 μg of purified cRNA were used for sense single-strand cDNA (ss-cDNA) synthesis, followed by RNase H digestion and ss-cDNA magnetic bead purification. Approximately 5.5 μg of ss-cDNA were fragmented using uracil DNA-glycosylase (10 U/μL) and apurinic/apyrimidinic endonuclease 1 (1.000 U/μL), and then labeled with biotin using terminal deoxynucleotidyl transferase (30 U/μL). From the hybridization cocktail, 200 μL of the obtained mixture were loaded into single human Clariom D 49-format array and incubated for 16 h in the Affymetrix GeneChip Hybridization Oven 645 at 45 °C, 60 rpm. Arrays were stained using an Affymetrix GeneChip Fluidics Station 450, according to the specific fluidics protocol (FS450_0001), and scanned with an Affymetrix GeneChip Scanner 3000 7G. Raw intensity CEL files generated by GeneChip™Command Console™ were imported into Transcriptome Analysis Console (TAC) 4.0 (Applied Biosystems) and CHP files were generated for gene-level analysis. Differentially expressed (DE) transcripts were identified by using TAC, with the following settings: Analysis Type: Expression Gene; Summarization Method: Gene Level - RMA. Gene-Level *P*-Value < 0.05 ANOVA Method: ebayes. Simultaneously, microarray data were also analyzed by MeV (Multi Experiment Viewer) v4.9.0 (http://mev.www.tm4.org, accessed on 19 July 2018) by applying Significance of Microarrays Analysis (SAM); paired and unpaired tests were performed among Δ*C*ts using a *p*-value based on 100 permutations; imputation engine: K-nearest neighbors (10 neighbors); false discovery rate (FDR) < 0.05. Results from paired and unpaired analyses were compared, selecting common transcripts for validation analysis.

### 4.4. Validation of Microarray Results Using Real-Time PCR

DE transcripts identified in the microarray analysis were validated in an independent cohort of 37 AD patients and 37 age- and sex-matched controls. PCR primers for the DE transcripts were designed using PrimerBlast (https://www.ncbi.nlm.nih.gov/tools/primer-blast/, accessed on 10 December 2018) (Table 7).

PCR reactions were performed using 50 ng RNA in input for each assay and Power SYBR^®^ Green RNA-to-CT™ 1-Step Kit (Thermo Fisher Scientific, Waltham, MA, USA), according to the manufacturer’s instructions. *GAPDH* (glyceraldehyde-3-phosphate dehydrogenase) and *RNU6* (RNA, U6 small nuclear 1) were used as endogenous controls. All reactions were performed on a 7900HT Fast Real-Time PCR System (Foster City, CA, USA). DE transcripts were identified using SDS RQ Manager 1.2 software (Foster City, CA, USA); by applying the 2^−ΔΔ*C*t^ method, and differential expression was expressed as RQ (Relative Quantity); RQ values < 1 were converted in fold change (FC) by applying the formula −1/RQ.

### 4.5. ROC Curve Analysis

To assess the potential diagnostic accuracy of DE transcripts, we computed ROC curves using SPSS 23 (IBM). For each ROC curve, generated by using expression values (Δ*C*ts), area under the curve (AUC), 95% confidence intervals (CIs) and *p*-value were calculated; the Youden method was applied to identify the optimal cut-off, with the associated sensitivity, specificity, accuracy, positive predictive value (PPV) and negative predictive values (NPV). In order to investigate if different combinations of DE transcripts could increase diagnostic accuracy, we also computed a ROC curve generated from a molecular signature of multiple transcripts: this multivariable ROC curve was computed considering all DE transcripts by building a binary logistic regression model through SPSS [60]. Statistical significance was established at a *p*-value < 0.05 for all ROC curves.

### 4.6. Expression Analysis in Peripheral Blood Mononuclear Cells

Expression of DE transcripts was also investigated in cells isolated from peripheral blood of 10 AD patients and 10 non-matched unaffected individuals included in the validation cohort. Peripheral blood mononuclear cells (PBMCs) from patients and controls were isolated from heparinized whole blood by gradient centrifugation over Ficoll-Hypaque solution (Ficoll Paque PLUS–GE Healthcare Life Sciences, Piscataway, NJ, USA). Lymphocytes were prepared from blood samples by using the Lympholyte^®^-H density gradient separation medium (Cedarlane, Burlington, NC, USA) according to the manufacturer’s instructions, with slight modifications. Briefly, the blood was diluted by adding an equal volume of complete RPMI 1640 medium (20% FBS, 1% penicillin/streptomycin, 1% L-glutammine, and 5% Phytohaemagglutinin). Two parts of diluted blood were added to one part of Lympholyte^®^-H. A centrifugation step (400× *g* for 25 min) was performed to separate the lymphocyte fraction from the whole blood; a well-defined lymphocyte layer appeared at the interface at the end of this step. The lymphocyte fraction was removed from the interface and transferred to a new centrifuge tube containing complete RPMI 1640 medium, followed by a centrifugation step (400× *g* for 10 min). After two additional washing steps, the isolated lymphocyte fraction was incubated in 25 cm^2^ culture flasks containing RPMI medium for 72 h at 37 °C and 5% CO_2_. Lymphocytes (4 × 10^6^) were then stored at −80 °C until use. Total RNA was isolated from PBMC pellets by using TRIzol (Thermo Fisher Scientific, Waltham, MA, USA), according to the manufacturer’s instructions. Expression analysis was performed by Real-Time PCR as described above, using 50 ng of RNA for each assay. ACTB (actin beta) and RNU6 were used as endogenous controls.

### 4.7. Statistical Analysis

Statistical analysis of expression data was performed using GraphPad Prism 8. PCR data (Δ*C*ts) were tested for normality of distributions (D’Agostino & Pearson omnibus normality test and Shapiro–Wilk normality test) and homogeneity of variance (F test); according to the results, parametric or non-parametric *t*-test was applied to evaluate statistically significant differences in transcript expression. Both paired (paired *t*-test or Wilcoxon test) and unpaired (homoscedastic or Welch corrected unpaired *t*-test, or Mann–Whitney test) analysis were performed.

To assess if age and sex represented confounding factors in this study, we followed a three-step procedure [61]: (1) the difference between AD patients and unaffected individuals in age and sex was evaluated through *t*-test/Mann–Whitney test (according to normality of distributions); (2) the association of sex/age with disease risk was evaluated using Binomial Logistic Regression; (3) the association of sex/age with DE transcript expression was evaluated by calculating Pearson/Spearman correlation coefficient (according to normality of distributions).

Correlation analysis was performed to evaluate the existence of relationships between plasma expression levels of DE transcripts (−Δ*C*ts) and cognitive decline of AD patients and unaffected individuals. After checking for normality of distributions, Pearson/Spearman correlation coefficient was calculated. Correlation *p*-values were corrected for multiple comparisons (Holm-Sidak method).

## 5. Conclusions

This study identified six transcripts (GS1-304P7.3, NONHSAT090268, TC0100011037, TC0400008478, TC1400008125, and *UBE2V1*) showing an altered expression in the plasma of mild-moderate AD patients compared to unaffected individuals. Given the need of new accurate blood biomarkers for AD diagnosis, these transcripts may be considered for further analyses in larger cohorts, also in combination with other biomarkers (including both Aβ-based biomarkers and miRNAs or other RNA molecules), with the aim of identifying specific RNA-based biomarkers to be eventually validated and introduced into clinical practice. Further studies enrolling patients with mild cognitive impairment would be useful to investigate if DE transcript expression may predict the severity of cognitive decline, potentially in an early phase of AD pathogenesis.

## Figures and Tables

**Table 1:** Real-Time PCR results. For each transcript, TAC ID, gene symbol (where available), median fold change and *p-*value (between brackets) are shown for each endogenous control (*GAPDH* and *RNU6*) and for both paired and unpaired analyses. Significant values are highlighted in bold. TAC: Transcriptome Analysis Console; *GAPDH*: glyceraldehyde-3-phosphate dehydrogenase; *RNU6*: RNA, U6 small nuclear 1.

TAC ID	Gene Symbol	*GAPDH*		*RNU6*	
		Paired	Unpaired	Paired	Unpaired
TC0100010930	GS1-304P7.3	**1.93 (0.027)**	**2.79 (0.008)**	**1.91 (0.043)**	**2.18 (0.04)**
TC0100011037		**−2.16 (0.045)**	**−2.58 (0.046)**	**−3.17 (0.006)**	**−3.21 (0.01)**
TC0100013007		−1.12 (0.63)	−1.21 (0.44)	1.09 (0.77)	1.04 (0.5)
TC0100015528		1.18 (0.86)	1.24 (0.87)	2.48 (0.84)	2 (0.83)
TC0100016418		−1.31 (0.52)	−1.31 (0.51)	−1.52 (0.64)	−2.07 (0.64)
TC0300007694	NONHSAT090268	**−2.74 (0.007)**	**−3.04 (0.006)**	**−1.71 (0.007)**	**−3.74 (0.008)**
TC0300013071	zyjeebu	−1.78 (0.71)	−2.36 (0.65)	−1.77 (0.83)	−1.44 (0.82)
TC0400008478		**−15.75 (0.016)**	**−11.22 (0.003)**	**−63.17 (0.016)**	**−7.04 (0.002)**
TC0500012139	peybleeby	−1.22 (0.8)	1.17 (0.74)	−1.33 (0.68)	1.12 (0.9)
TC0600007285	*HIST1H2AE*	1.06 (0.98)	1.57 (0.98)	1.36 (0.48)	−1.78 (0.47)
TC0600007784		−1.23 (0.99)	−1.23 (0.32)	−1.31 (0.79)	1.48 (0.81)
TC0800009993	blawker	−1.15 (0.91)	2.43 (0.88)	1.47 (0.72)	1.76 (0.66)
TC1000010059	NONHSAT011783	1.19 (0.37)	1.85 (0.19)	1.3 (0.88)	−1.35 (0.91)
TC1200011311	*NAP1L1*	1.07 (0.99)	4.21 (0.85)	1.17 (0.9)	3.9 (0.85)
TC1400008125		**−1.55 (0.037)**	**−2.25 (0.032)**	**−1.62 (0.021)**	**−2.36 (0.042)**
TC1600007870		1.08 (0.99)	−2.04 (0.99)	−1.35 (0.95)	1.02 (0.94)
TC1600010293	swoyry	−1.27 (0.3)	−1.76 (0.5)	−1.78 (0.06)	−3.32 (0.09)
TC1900010363		−2.57 (0.67)	−2.46 (0.71)	−1.59 (0.63)	1.02 (0.56)
TC2000010025	*UBE2V1*	**−1.37 (0.037)**	**−1.63 (0.048)**	**−1.98 (0.028)**	**−2.1 (0.045)**

**Table 2:** Results of univariable ROC (receiver operating characteristic) curve analysis. For each transcript, the *p*-value of the curve, the area under the curve (AUC), its standard error (Std error), and the 95% confidence intervals (CIs) are shown; the Youden index was also calculated to identify the optimal cut-off, for which sensitivity, specificity, accuracy, positive predictive value (PPV) and negative predictive value (NPV) are shown. Significant values are highlighted in bold.

DE Transcript	AUC	Std Error	*P*-Value	95% CIs	Cut-Off	Sensitivity	Specificity	Accuracy	PPV	NPV
GS1-304P7.3	**0.722**	**0.074**	**0.008**	**0.578–0.867**	**1.85**	**0.75**	**0.71**	**0.73**	**0.72**	**0.74**
NONHSAT090268	**0.75**3	**0.069**	**0.002**	**0.618–0.887**	**3.12**	**0.68**	**0.81**	**0.74**	**0.77**	**0.71**
TC0100011037	**0.716**	**0.072**	**0.006**	**0.576–0.865**	**2.66**	**0.67**	**0.74**	**0.7**	**0.72**	**0.69**
TC0400008478	**0.803**	**0.078**	**0.001**	**0.65–0.957**	**1.97**	**0.95**	**0.68**	**0.82**	**0.75**	**0.93**
TC1400008125	0.644	0.076	0.064	0.494–0.794	2.07	0.5	0.89	0.7	0.82	0.64
*UBE2V1*	**0.637**	**0.065**	**0.045**	**0.51–0.765**	**1.95**	**0.78**	**0.47**	**0.63**	**0.6**	**0.68**

**Table 3:** Features of the ROC curve computed on all DE transcripts. The *p*-value of the curve, the AUC, its standard error (Std error), and the 95% CIs are shown; sensitivity, specificity, accuracy, PPV and NPV are also shown. Significant values are highlighted in bold.

Transcript Signature	*P*-Value	AUC	Std Error	95% CIs	Sensitivity	Specificity	Accuracy	PPV	NPV
GS1-304P7.3, NONHSAT090268, TC0100011037, TC0400008478, TC1400008125, *UBE2V1*	**0.00007**	**0.772**	**0.054**	**0.667–0.878**	**0.64**	**0.72**	**0.68**	**0.7**	**0.67**

**Table 4:** Expression analysis performed on PBMCs. For each transcript, median fold change and *p*-value (between brackets) are shown for each endogenous control (*ACTB* and *RNU6*). *ACTB*: actin beta.

Transcript	*ACTB*	*RNU6*
GS1-304P7.3	−2.55 (0.37)	−1.42 (0.51)
NONHSAT090268	−2.51 (0.36)	−1.41 (0.36)
TC0100011037	−2.54 (0.37)	−1.42 (0.51)
TC0400008478	−2.49 (0.39)	−1.39 (0.54)
TC1400008125	−3.01 (0.39)	−1.68 (0.34)
*UBE2V1*	−1.52 (0.53)	1.17 (0.47)

**Table 5:** Correlation analysis between plasma levels of DE transcripts and clinicopathological parameters. For each transcript/parameter pair, the *r*-value (Pearson or Spearman correlation coefficient, according to normality of distributions) and the *p*-value corrected for multiple comparison (between brackets) are shown. Significant values are highlighted in bold. MMSE: Mini Mental State Examination.

DE Transcript	MMSE T0	Delta MMSE/Month
GS1-304P7.3	**−0.36 (0.024)**	0.29 (0.17)
NONHSAT090268	**0.38 (0.012)**	0.14 (0.51)
TC0100011037	**0.32 (0.03)**	0.16 (0.42)
TC0400008478	**0.48 (0.02)**	0.33 (0.13)
TC1400008125	0.02 (0.85)	−0.1 (0.85)
*UBE2V1*	−0.05 (0.67)	−0.14 (0.65)

**Table 6:** Clinicopathological features of AD patients and unaffected individuals enrolled for this study. Data are presented as average ± standard deviation. AD: Alzheimer’s disease; N/A: not available.

	Sex (M/F)	Age	MMSE T0	MMSE T1	Delta MMSE/Month
AD	17/25	74.51 ± 6.95	18.58 ± 5.4	14.54 ± 6.01	−0.32 ± 0.21
CTRL	17/25	73.72 ± 7.34	29.64 ± 0.48	N/A	N/A

**Table 7:** PCR primers used for Real-Time PCR validation assays.

Transcript	Forward Primer	Reverse Primer
*ACTB*	GAGCACAGAGCCTCGCCTTT	GAGCGCGGCGATATCATCA
blawker	AACCTGGGGCTGGTAAAGGTA	TGTGCTGCTGTTTTGGTAGTCA
*GAPDH*	TGCACCACCAACTGCTTAGC	GGCATGGACTGTGGTCATGAG
GS1-304P7.3	CCAGGGACCCAGAACAGATAGT	GGTCCCTAGACACTGACGAAATC
*HIST1H2AE*	AAGAAGACGGAGAGCCACCA	GACTCGGGATCACTGACGGA
*NAP1L1*	GGCAGACATTGACAACAAAGAAC	AGCTGACGTGCTTTGAG
NONHSAT011783	TTGGTGATAGAAAAGGGCTGAAGT	GTGGCTCTCTCGGACAATGC
NONHSAT090268	TCTGGCCTTACCACCTCCTTT	GAGTGGAAATGACAACTTGATGCTC
peybleeby	ATGGTACAGGGTGATGGGCT	GCACCCTCCCCCACCTAATA
*RNU6*	CTCGCTTCGGCAGCACA	AACGCTTCACGAATTTGCGT
swoyry	TTCCTGGATGAGTGTCCTGGG	TATGGTGAGGGCAGTTGTCTCT
TC0100011037	TTGAGTTAGCGAGTGGGGAGA	TGCAAATCTGGGGTTTGACCT
TC0100013007	GGAAAGTCTCTGAGGAAACAGCA	GAGTAACCCATGCCTGCTCC
TC0100015528	CACCTAGCCATCCCCACTGA	TTCTTTTGCTTGTGGCGTGC
TC0100016418	TGACACAGGATAAGCGCAACA	CCCCCTTTACCTTCCTTGAGC
TC0400008478	GCTCTGGAAAACCACAGGGTC	ATAGATCTGTGGCCAGGTGAGG
TC0600007784	CCTGATCCATGCCTAGAGGTTGA	TGGAGAAACTCAATGACACCAGAAG
TC1400008125	AGTTGCAAGAACGAACGGGA	CATAGGCTGGCTTGTGGAGG
TC1600007870	CGCCTCTACCTCCAGTGTGA	GGCCAGAGTGGAGCCATGTA
TC1900010363	AGGAGGAGACACACCCAAAAGA	GAATGCTTTTTAAGGGTGCGAGC
*UBE2V1*	GTTGTCCTGCAAGAGCTTCG	TGTAACACTGTCCTTCGGGC
zyjeebu	TGTTGGCACAGTCCGTTGTC	CTCCCCTAACCTCACAGGCA

## Abbreviations

AD	Alzheimer’s disease
ADAMTS9	ADAM metallopeptidase with thrombospondin type 1 motif 9
ADAMTS9-AS2	ADAMTS9 antisense RNA 2
ARSJ	arylsulfatase family member J
AUC	area under the curve
Aβ	amyloid beta
circRNAs	circular RNAs
CIs	confidence intervals
CNS	central nervous system
CSF	cerebrospinal fluid
CTRL	Controls
DE	differentially expressed
GAPDH	glyceraldehyde-3-phosphate dehydrogenase
lncRNAs	long non-coding RNAs
miRNAs	microRNAs
MMSE	Mini Mental State Examination
ncRNAs	non-coding RNAs
NPV	negative predictive value
PBMCs	peripheral blood mononuclear cells
PDC	Phosducin
PDC-AS1	PDC antisense RNA 1
PPV	positive predictive value
PSD95	postsynaptic density-95
PTGS2	prostaglandin-endoperoxide synthase 2
RNU6	RNA, U6 small nuclear 1
ROC	receiver operating characteristic
RPL23A	ribosomal protein L23a
UBE2V1	ubiquitin conjugating enzyme E2 V1)
UGT8	UDP glycosyltransferase 8
